# Strengthening the research to policy and practice interface: exploring strategies used by research organisations working on sexual and reproductive health and HIV/AIDS

**DOI:** 10.1186/1478-4505-9-S1-S2

**Published:** 2011-06-16

**Authors:** Sally Theobald, Olivia Tulloch, Joanna Crichton, Kate Hawkins, Eliya Zulu, Philippe Mayaud, Justin Parkhurst, Alan Whiteside, Hilary Standing

**Affiliations:** 1 International Health Research Group, Liverpool School of Tropical Medicine, Pembroke Place, Liverpool, L3 5QA, UK; 2 African Population and Health Research Center, PO Box 10787, 00100 GPO, Nairobi, Kenya Current address: School of Social and Community Medicine, University of Bristol, Canynge Hall, 39 Whatley Road, Bristol, BS8 2PS, UK; 3 Knowledge Technology and Society Team, Institute of Development Studies at the University of Sussex, Brighton BN1 9RE, UK; 4 African Institute for Development Policy (AFIDEP), P. O. Box 14688-00800, Westlands, Nairobi, Kenya; 5 Infectious Diseases & Reproductive Health, London School of Hygiene and Tropical Medicine, Room 248a, Keppel St, London WC1E 7HT, UK; 6 Health Policy Unit, London School of Hygiene and Tropical Medicine, 15-17 Tavistock Place, London, WC1H 9SH, UK; 7 Health Economics & HIV/AIDS Research Division, University of Kwazulu-Natal, South Africa

## Abstract

This commentary introduces the HARPS supplement on getting research into policy and practice in sexual and reproductive health (SRH). The papers in this supplement have been produced by the Sexual Health and HIV Evidence into Practice (SHHEP) collaboration of international research, practitioner and advocacy organizations based in research programmes funded by the UK Department for International Development.

The commentary describes the increasing interest from research and communication practitioners, policy makers and funders in expanding the impact of research on policy and practice. It notes the need for contextually embedded understanding of ways to engage multiple stakeholders in the politicized, sensitive and often contested arenas of sexual and reproductive health. The commentary then introduces the papers under their respective themes: (1) The theory and practice of research engagement (two global papers); (2) Applying policy analysis to explore the role of research evidence in SRH and HIV/AIDS policy (two papers with examples from Ghana, Malawi, Uganda and Zambia); (3) Strategies and methodologies for engagement (five papers on Kenya, South Africa, Ghana, Tanzania and Swaziland respectively); (4) Advocacy and engagement to influence attitudes on controversial elements of sexual health (two papers, Bangladesh and global); and (5) Institutional approaches to inter-sectoral engagement for action and strengthening research communications (two papers, Ghana and global).

The papers illustrate the many forms research impact can take in the field of sexual and reproductive health. This includes *discursive changes* through carving out legitimate spaces for public debate; *content changes* such as contributing to changing laws and practices, *procedural changes* such as influencing how data on SRH are collected, and behavioural changes through partnerships with civil society actors such as advocacy groups and journalists.

The contributions to this supplement provide a body of critical analysis of communication and engagement strategies across the spectrum of SRH and HIV/AIDS research through the testing of different models for the research-to-policy interface. They provide new insights on how researchers and communication specialists can respond to changing policy climates to create windows of opportunity for influence.

## Commentary

Interest in research uptake and research engagement is growing. The impetus for this growth comes from multiple constituencies. First, in the context of addressing the high levels of morbidity and mortality associated with HIV and sexual and reproductive health (SRH) in resource poor contexts researchers, communication specialists and donors may feel a moral and ethical imperative to try to ensure that policy and practice draws on the best research available. In some countries civil society and activist groups have been active in calling governments to account to provide the best treatment available, as exemplified by the Treatment Action Campaign’s efforts to ensure provision of antiretroviral drugs in South Africa.

Second, research impact and communications are on the agenda of many funders, who are keen to show the impact of their investment and justify this to their own constituencies or tax payers. For instance ‘research impact’ carries a third of marks in proposal assessment for European Union funded research proposals. In the UK, the Economic and Social Research Council has recently appointed a strategic adviser for research and impact and the UK Department for International Development (DFID) research programmes have stipulated that research uptake activities are integrated into research programmes.

This supplement presents conceptual and empirical analyses of the research-policy interface. It draws on work carried out by researchers and communication specialists from four Research Programme Consortia (RPC) funded by DFID which focus on SRH and HIV/AIDS. The RPCs have been active in Sub-Saharan (South Africa, Malawi, Uganda, Zambia, Tanzania, Kenya, Ghana) and Asia (Bangladesh and India) as well as the UK. The articles were produced as part of the Sexual Health and HIV Evidence into Practice (SHHEP) initiative, which is a collaboration across these four RPCs. This work was prompted by a desire to learn about and improve research to policy and practice processes, and to explore how investment of time and resources in this area had an impact on the work of the RPCs.

A key challenge in understanding research-policy links is the multiplicity of contexts and variables that affect the relationships between the two sectors. This supplement focuses on the research-policy interface in one broad area – that of SRH and HIV/AIDS – enabling detailed discussion of the issues at stake in policy engagement in one, albeit fast moving and fluid area of the health sector that involves multiple stakeholders working at different levels. These issues can be highly politicised, sensitive and challenging and the messages and processes through which to engage with diverse stakeholders in different contexts need careful consideration.

The articles in this supplement provide critical analysis of communication and engagement strategies across the spectrum of SRH and HIV/AIDS research through reflective application and the testing of different models for the research-to-policy interface. In many contexts, SRH issues are conceptualised by policy makers and practitioners as low priority, low profile, controversial and unpopular. HIV and AIDS on the other hand, following long and sustained advocacy and campaigning has risen up national and international policy agendas. We bring new insights on research communication by increasing understanding of how researchers and communication specialists worked on different SRH and HIV issues and with different research methodologies, responding to changing policy climates to create windows of opportunity for influence. These insights also have implications for other health issues and beyond the SRH and HIV.

There is need for increased reflection and experimentation with research communication techniques to enable academics and communication specialists to be more strategic about the tools and approaches they use to target particular audiences. The articles represent a broad range of strategies developed and applied by SHHEP researchers and communication specialists to influence different actors (for example policy makers, practitioners and the media). They show that a deep understanding of context is critical in research engagement approaches, as is local credibility and trust and capacity to sustain multi-level relationships over long periods.

The papers in this supplement address the following questions:

1. How do SRH and HIV/AIDS research organisations describe their policy influencing aims, and who are the policy makers they seek to influence?

2. What influencing strategies and approaches are used by SRH, HIV and AIDS research organisations and how are these shaped by methodology, context and subject area?

3. What are the different ways in which research evidence is strategically framed in order to maximise impact?

4. What strategies do research organisations use to track the impact of their work?

5. From the perspective of research organisations, what models and conceptual frameworks are helpful in informing and framing research engagement?

The papers are organised according to five themes:

### Theme 1. The theory and practice of research engagement

Two articles (Sumner et al [1]; Crichton and Theobald [2]) frame the supplement by reviewing the theory and practice of research engagement. Sumner et al [1] synthesise the diversity of theoretical approaches to research uptake, illustrating their uses in supporting and conceptualising research engagement in different fields within SRH and HIV/AIDS. They present an analytical framework to illustrate how the opportunities for research to shape policy and practice are driven by an interaction between policy ideas, policy actors and political context. In later sections, this analytical framework is applied to selected articles in the supplement to show how they interact in the current context on a specific SRH issue (for example, cotrimoxazole prophylaxis, Gender Based Violence) and to provide examples of interventions to increase the probability of research impact in SRH and HIV/AIDS policy.

Crichton and Theobald’s paper [2] uses in-depth interviews with researchers and communications specialists working within the four DFID funded RPCs to explore their understandings, motivation, dilemmas and framing of research engagement in SRH and HIV/AIDS research. They adapt the Overseas Development Institute’s ‘RAPID’ (research and policy in development) framework [3] to include an explicit focus on research actors’ positionality and use this to interrogate and present their analysis. They argue that the attributes, skills, position and disciplinary outlooks of researchers and communications specialists, and their perceptions about the role of research evidence in policy and practice, feed into their approach to influence. They suggest rather than having a blueprint approach to intensification and diversification of policy engagement for research actors and institutions, a variety of research influencing goals and engagement approaches is desirable. The articles that follow illustrate this diversity of approaches.

### Theme 2: Applying policy analysis to explore the role of research evidence in SRH and HIV/AIDS policy

The two articles in this theme use policy analysis to understand the role of research evidence in national and international SRH and HIV/AIDS policy formation. In a case-study from Ghana, Burris et al [4] examine the process through which research evidence related to the interrelations between Herpes simplex virus type-2 (HSV-2) infection and HIV, influenced treatment policies and guidelines at the international level and what were the mechanisms of international-to-national policy transfer. The analysis highlights the pivotal role of ‘policy networks’ and policy entrepreneurs in shaping research uptake. Policy networks were formed either formally at an international level (WHO) or informally (within Ghana). The case-study illustrates that within sexual health policy networks, there is often a single individual or ‘policy entrepreneur’ who generates the momentum for policy change. Donor influence can also be critical and was cited as the single strongest impetus or impediment to policy change in Ghana.

A comparative policy analysis by Hutchinson et al [5] discusses how national policies respond to emerging research evidence on cotrimoxazole as an inexpensive and highly efficacious preventative intervention in HIV infected individuals in Malawi, Uganda and Zambia. They apply the RAPID framework to explore differences in policy formulation, highlighting the critical roles of the context in which evidence is considered, the nature and understanding of that evidence, and the links between key knowledge brokers and policy makers. Operational research evidence seems to have been taken up quickly, while evidence from randomised controlled trials was not necessarily translated into policy so swiftly if the issue was not framed as policy relevant, or there was not a policy window in which change could occur. As in all settings, the role of powerful policy entrepreneurs was critical and instrumental in pushing cotrimoxazole preventive therapy onto the agenda. The links between different research and policy actors are important, and the authors argue that overlaps between different networks are central in facilitating knowledge transfer.

### Theme 3: Strategies and methodologies for engagement

This theme focuses on different strategies and methodologies for research engagement. These include developing meaningful relationships and exchange with intermediaries (Oronje et al on working with the media in Kenya [6]) and with community and trial participants (Delany-Moretlwe et al in South Africa [7]). Methodologies include impact analysis (Henry and Whiteside on the impact of a report on HIV in Swaziland [8]), a case-study analysis (Tulloch et al on the lessons learnt from four case-studies on research engagement in Sub-Saharan Africa [9]) and developing a “boundary organisation” to enable exchange across and between research and policy actor institutions (Drimie & Quinlan’s paper on HIV and nutrition in South Africa [10]).

Oronje et al [6] illustrate practical approaches to overcoming mutual distrust between researchers and media specialists and building the technical capacities of both camps in enhancing the use of research in the media. Using a case-study analysis of multiple strategies for engaging with the media they show how one international research organisation (the Kenya-based African Population and Health Research Center – APHRC) has sought to develop trusting relationships between researchers and the media. This involved structured meetings, informal lunch meetings, mutual training and incentives for journalists to cover SRH issues in the press (through competitions and prizes for best coverage) and partnership with the producers of a popular Kenyan soap opera – “Makutano Junction” – to ensure research informed plot development and raise awareness, tackle stigma and promote information about underused SRH services. APHRC also worked with the producers to support viewers’ requests for further information through sending support resources by post or SMS text messages. The authors recognized that communicating research through the mass media is important in moving research to policy and action, contributing to specific Kenyan policy decisions, addressing the culture of silence on SRH issues, countering stigma and promoting access to information. The impact however, is not easy to measure and many journalists lack interest and the capacity to produce high quality coverage of research findings.

The article by Delany-Moretlwe et al [7] provides an interesting illustration of the importance of the political context in which evidence is considered – in this case how the apartheid history of South Africa left a legacy of suspicion and mistrust of research. Investment in communicating research results and building partnerships with communities and trial participants has been essential for building trust. They explore the process of conducting and communicating randomised controlled trials to test the causal relationship between HSV-2 infection and HIV transmission. The paper highlights the process of developing a communication plan, and ongoing dialogue and relationship building with different communities through Community Advisory Boards and Community Based Organisations. The personal touch can be critical; they report that staff, trial participants and community members were heavily invested in a positive outcome of the research and this illustrates how important trials can be, at a personal level, in terms of the hope they represent in the fight against HIV. All study participants were informed of the results of the trials by SMS text messages before media announcements. The investments made in community engagement brought knowledge to communities and interest in research, which created an enabling environment for future SRH and HIV/AIDS research.

Henry and Whiteside’s paper [8] shows how impact assessment can be of real value in understanding the research-to-policy interface, and ultimately the impact of their research. They conducted an impact assessment of the report ‘*Reviewing ‘Emergencies’ for Swaziland: Shifting the Paradigm in a New Era*’ which aimed to bring the devastating effect of HIV on all aspects of Swazi life to the attention of local policy makers and international donors. An assessment of the impact of the report was commissioned to understand how, where and why it had (and had not) made an impact. This article highlights the importance of finding terminologies that stick and have impact in the context of AIDS in Swaziland as a ‘long-term emergency.’ The assessment showed the importance of knowing and being embedded in context, and the credibility of the message bearer.

Tulloch et al [9] use case-study analysis to explore the enablers and constraints of how research is translated into policy *and* practice in the cases of male circumcision in South Africa and Tanzania; gender based violence, and maternal syphilis screening in Ghana. This case-study analysis highlights the importance of looking critically at practice within different settings and not assuming that policy change will automatically result in improved implementation. Lessons learnt include the need for researchers and communication specialists to work at developing networks of actors across the policy and practice continuum, and designing long-term communications strategies which are appropriate to a range of specific technical, political and cultural contexts.

Drimie and Quinlan [10] deploy the concept of a ‘boundary organisation’ to frame and analyse research engagement. A boundary organisation is one that cuts across research and policy maker communities to support co-production – of research and the application of research – in policy and practice. The authors discuss the Health Economics and HIV/AIDS Research Division’s (HEARD) ‘boundary organisation’ role of leading the Regional Network on AIDS, Livelihoods and Food Security (RENEWAL). Key challenges include the politics of political engagement and the time constraints of policy makers, nurturing policy champions, and maintaining the integrity of the interactive research agenda. The RENEWAL experience shows that boundary organisations need to be simultaneously flexible and persistent and that the gradual strengthening of networks needs to bring in diverse constituencies to support legitimacy and engender trust.

### Theme 4: Advocacy and engagement to influence attitudes on controversial elements of sexual health

Two commentaries offer accounts of reflective learning at institutional level which highlighted lessons learnt in advocacy and engagement in relation to influencing attitudes on controversial elements of sexual health. Rashid et al [11] show that it is possible to create a public space and dialogue on sexuality and rights in a conservative and challenging environment like Bangladesh by bringing together a diverse group of stakeholders – lesbian, gay, bisexual and transsexual groups, research, academics, NGO professionals, health providers, journalists and policymakers – to successfully challenge representations of sexuality in the public arena. This can be done by assessing strategic opportunities and building sustained alliances and partnerships with media, activists, academics and sexual minority group coalitions for long term change in attitudes, behaviour and legal frameworks. In assessing the impact of this kind of initiative, the authors argue that attention should be paid to understanding the role of interim outcomes as a marker of research influence in a complex environment.

Knerr and Philpott’s engaging article [12] commentary describes the journey of one organisation - The Pleasure Project - to bridge the worlds of academia, public health and the sex industry to improve sexual health outcomes. The Pleasure Project argues for greater focus on effective sexual health interventions that incorporate pleasure as a key component, with the continuing aim of bridging the sexual health–pleasure divide. Knerr and Philpott [12] note that the public health response to HIV has been and continues to be overwhelmingly focused on the negative outcomes of sex. However evidence shows that eroticisation of safer sex and inclusion of pleasure in sex education narratives can be effective encouragements to safer sex. The authors analyse how The Pleasure Project communicates messages about eroticising safer sex to diverse audiences and assess how well these messages have worked. Key lessons are the need to challenge health professionals’ pre-conceptions and discomfort with issues related to sex and sexuality and to combine all levels of evidence – from the experimental to the anecdotal – and bringing experiential knowledge from the sex industry to bridge the health–pleasure divide.

### Theme 5: Institutional approaches to intersectoral engagement for action and strengthening research communications

Two commentaries synthesise institutional approaches to intersectoral engagement for action and capacity strengthening. Gyapong et al’s commentary piece [13] is a critical reflection on the role of the Research and Development Division (RDD) of the Ghana Health Service in fostering inter-sectoral action to support Ghanaian Orphans and Vulnerable Children (OVC). Gyapong et al [13] argue that the multifaceted nature of HIV/AIDS requires new partnerships with multiple stakeholders. Through being situated within a government department, RDD has good access to directors and programme managers. The paper shows how this institutional positionality has enabled collaboration with senior managers within the Ministry of Health, Department of Social Welfare and other key stakeholders to take forward research and action on OVC. The paper reflects on the opportunities and challenges of multiple stakeholder engagement throughout the cycle of research: from agenda setting to discussions on policy relevance.

South’s short commentary piece [14] summarises lessons learnt from the four DFID funded RPCs represented in this supplement. DFID’s stipulation of investment in research uptake (minimum of 10% in the previous round of RPCs) has strengthened the legitimacy of communications activities. South [14] argues for the importance of supporting communication specialists, including financial support to enable activities to take place, training, access to resources and expertise, and being part of a community of people working to promote the use of research findings. She outlines the work of and learning from a Community of Practice, which served to link communications specialists working on one RPC – Evidence for Action. Key here is the importance – for all of those involved – of a sense of belonging to a community which is supportive of ideas and action.

Young and Mendizabal [15] have developed a categorisation of the range of impacts research can have. They categorise impact as: attitudinal (changes in the perceptions of key stakeholders); discursive (changes in language usage); content (changes in written policy); procedural (changing how something is done); and behavioural changes (sustainable changes in the way something is perceived or approached). We present an adapted version of this framework below to characterise the influences seen in the field of SRH and HIV/AIDS illustrated in this supplement:

In linking research to policy and practice, these articles illustrate the importance of:

1. Undertaking reflective assessments of the policy relevance of research evidence, its scope and limitations within a particular context and the ethical implications of communicating the research

2. Carrying out strategic scoping of opportunities and levers for influence through analysis of the policy context, actors and processes, including the political or cultural acceptability of research findings

3. Assessing the nature of the research evidence and consulting with other key actors on how best to frame it in ways that increase local decision makers’ receptivity.

4. Keeping communications strategies flexible, innovative, jargon free and relevant to research institutions’ objectives to keep them effective.

5. Being aware of the broad range of research impacts (Table 1)

**Table 1:** Categorising Research Impact

Broad category of research influence	Specific areas in SRH, HIV and AIDS sector	Illustrative examples
Discursive changes	Change discourse	Opening new public spaces/discourses on representations of sexuality in Bangladesh (Rashid et al [11]) Challenging key academic or policy discourses, for example on health economics and on Children and HIV, and gender and masculinities (Crichton and Theobald [3])

Content changes	Change laws/policies	Change in law to exempt survivors of gender-based violence from paying medical costs (Tulloch et al [9]) Research to analyse the process of uptake of Cotrimoxazole preventive therapy in Zambia, Uganda and Malawi and draw out lessons on policy change (Hutchinson et al [5])

Procedural changes	Change how health-related government ministries or agencies analyse their data on service delivery	Gender disaggregated data and equity analysis of ART data now part of MoH processes (REACH Trust, Malawi) (Crichton and Theobald [3])

Behavioural changes	Raise awareness of and access to research and capacity to understand it.	Media engagement and media capacity building: Raising awareness about health and rights through an entertaining and engaging drama – Makutano Junction (Oronje et al [6])
	
	Support attitudinal change	The Pleasure Project’s global mapping of pleasure used research to promote sexy safe sex (Knerr & Philpott [12])
	
	Build national capacity to carry out research and identify the policy implications	Research was undertaken on OVC within the Ministry of Health and links developed with other government departments (Gyapong et al [13])

The supplement shows the importance of coupling traditional research communications, for example, peer reviewed written work and face-to-face meetings with decision makers, with techniques that meet policy makers’, the media and communities’ needs for accessible and timely messaging and engagement. The supplement also illustrates a paradigm shift away from traditional dissemination at the end of research projects to fostering ongoing long-term engagement with different stakeholders throughout the research cycle. We illustrate these trends with case-studies focusing on sexual and reproductive health, HIV and AIDS but this paradigm shift and its implications are of broad relevance to health research in general. The supplement demonstrates that where health policy issues are highly politicised or controversial in a given context, or neglected by policy makers, this increases the importance of effective policy engagement strategies. There is need for researchers, communications and advocacy practitioners to strategise together to identify the communications objectives and most appropriate approaches for particular research projects. This requires sufficient resources, continual capacity building and ongoing critical review of research uptake strategies.

## Competing interests

The authors declare that they have no competing interests.
